# Field data on Vegetation Structure and Effects of Human Use of the Dambos Ecosystem in Northern Mozambique

**DOI:** 10.1016/j.dib.2019.104454

**Published:** 2019-09-12

**Authors:** Aires Afonso Mbanze, Amade Martins Mário, Rui Rivaes, Ana I. Ribeiro-Barros, Natasha Sofia Ribeiro

**Affiliations:** a Universidade Lúrio, Faculty of Agricultural Sciences (FCA), Sanga University Campus, Niassa Province, Mozambique; b Universidade de Lisboa, Instituto Superior de Agronomia (ISA), Linking Landscape, Environment, Agriculture and Food (LEAF), Tapada da Ajuda, P.O.Box, 1349-017, Lisbon, Portugal; c Universidade Nova de Lisboa, Nova School of Business and Economics, Campus de Carcavelos, Rua da Holanda 1, P.O.Box 2775-405, Lisbon, Portugal; d Technical Institute of Ecotourism Armando Emílio Guebuza, Marrupa District, Niassa Province, Mozambique; e Universidade de Lisboa, Instituto Superior de Agronomia (ISA), Forest Research Centre (CEF), Tapada da Ajuda, P.O.Box, 1349-017, Lisbon, Portugal; f Eduardo Mondlane University, Faculty of Agronomy and Forest Engineering, Av. J. Nyerere 3453/Campus Universitário Principal, Maputo, Mozambique

**Keywords:** Dambos ecosystem, Horizontal and vertical structure, Transect lines and vegetation survey

## Abstract

The data content of this paper is related to the original research article entitled *“Vegetation Structure and Effects of Human Use of the Dambo Ecosystem in Northern Mozambique”* that was published in the *Global Ecology and Conservation*. Woody and grass vegetation was inventoried in the dambos wetlands of the Niassa National Reserve (NNR), the largest Protected Area (PA) in Mozambique and the third largest in Africa. The six dambos assessed were selected through Google Earth, MODIS satellite images and exploratory field visits. The selected dambos were surveyed using a two-stage systematic sampling procedure in which woody vegetation was inventoried by means of transects, and the grass was inventoried using quadratic sub-plots laid down within the transects. The woody vegetation survey included the identification of all individuals to the species level, measurement of total height and diameter at breast height (DBH). The grass vegetation survey consisted of measurement of the total height and species identification within sub-plots. Woody vegetation data in this article includes also estimation of total richness, absolute and relative abundance, dominance, frequency, species volume and successional stage of each species in the vertical structure. Estimation of richness and absolute dominance is also presented for the grass vegetation.

**Table:** Specifications Table

Subject area	*Ecology*
More specific subject area	*Biodiversity conservation*
Type of data	*Table, Excel files and figure*
How data was acquired	*Field Survey*
Data format	*Analyzed and Raw data*
Experimental factors	*The main criteria that was used to select the dambos sampled were the following: predominance of grass vegetation (canopy cover less than 20%); Location closer to the* Mbatamila main office and seasonal or permanent water dambos.
Experimental features	*Data were collected using a two-stage systematic sampling procedure. In the first stage, height and diameters of tree and shrub vegetation was collected in main transects of 0.*1 ha*, after its identification. In a second stage, grass vegetation was counted, height and collected for later identification in six square subplots of 0.25m*^*2*^*(50 × 50 cm), established within the main transects.*
Data source location	*Niassa National Reserve, Mecula district, Mozambique.* *Latitude:* 12°38′48.67″S and 11°27′05.83″S *& Longitude:* 36°25′21.16″E and 38°30′23.74″E
Data accessibility	*All data are with this article*
Related research article	Author's name: Aires Afonso Mbanze, Amade Martins Mário, Rui Rivaes, Ana I. Ribeiro-Barros and Natasha Sofia Ribeiro Title: “*Vegetation Structure and Effects of Human Use of the Dambo Ecosystem in Northern Mozambique”* Journal: Global Ecology and Conservation DOI: https://doi.org/10.1016/j.gecco.2019.e00704

**Table:** 

**Value of the Data** • Data can be used to assess biodiversity losses, species threats, fragmentation and degradation of dambos wetland caused by anthropogenic disturbances mainly in the context of climate change and other threats; • Data can serve as a starting point for comprehensive research on comparison of vegetation patterns among dambos wetlands in African savannas; • Data can serve as the baseline for longitudinal and panel data studies to help decision-makers and other conservation experts to draft conservation guidelines; • Data can also be used in multidisciplinary studies for advanced analysis and simulation of different scenarios

## 1 Data

Information of the grass species, that includes species richness and absolute (Ab) dominance in each dambo and the whole ecosystem of dambos assessed, is provided in the Table 1. Table 2 presents detailed information related to the species and families of woody vegetation. Woody vegetation data, includes estimation of absolute and relative abundance (AbandAr), dominance (DaandDr), frequency (FaandFr) and volume and representativeness of each species in the vertical strata. Importance Value Index (IVI), was also computed in order to have a broader picture of the position of each species in the structure of the dambos [1], [2].

**Table 1:** Species richness and absolute dominance of the grass vegetation in the dambos. Species richness that is the total number of species assessed in each dambo is in the last line. The total in the lines, is the number of individuals per species, while in the last column, is the total number of individual grass of each dambo.

Dambos								
N^o^	Species	1	2	3	4	5	6	Total
1	*Alloteropsis semialata*	0	0	0	0	0	16	16
2	*Andropogon appendiculatus*	0	0	0	0	0	15	15
3	*Andropogon eucomus*	182	139	435	253	347	25	1381
4	*Andropogon gayanus*	133	99	249	26	49	154	710
5	*Andropogon huillensis*	0	28	0	44	88	0	160
6	*Andropogon schirensis*	0	0	0	0	0	13	13
7	*Anthephora pubescens*	0	9	0	34	22	0	65
8	*Aristida adscensionis*	98	37	38	141	29	74	417
9	*Aristida canescens*	0	0	0	0	0	12	12
10	*Aristida congesta*	17	28	51	8	0	78	182
11	*Aristida diffusa*	0	0	0	0	0	8	8
12	*Aristida junciformis*	122	0	0	0	0	296	418
13	*Aristida meridionalis*	0	0	0	0	0	24	24
14	*Aristida stipitata*	0	30	0	0	0	0	30
15	*Cenchrus ciliaris*	0	0	0	46	0	156	202
16	*Chrysopogon serrulatus*	28	0	0	21	0	71	120
17	*Ctenium concinnum*	0	0	4	0	0	0	4
18	*Cymbopogon excavatus*	19	0	0	0	0	28	47
19	*Cymbopogon plurinodis*	0	0	0	6	0	20	26
20	*Cymbopogon validus*	0	73	0	0	14	38	125
21	*Digitaria eriantha*	13	0	0	0	0	65	78
22	*Digitaria monodactyla*	1	0	0	19	27	83	130
23	*Ehrharta erecta*	6	6	0	0	0	0	12
24	*Elionurus muticus*	0	0	0	0	0	12	12
25	*Emarthria altissima*	0	0	0	0	0	61	61
26	*Enteropogon macrostachyus*	7	7	0	29	13	21	77
27	*Eragrostis capensis*	0	0	0	24	0	0	24
28	*Eragrostis ciliaris*	44	0	0	0	0	0	44
29	*Eragrostis pseudosclerantha*	0	7	0	11	0	0	18
30	*Eragrostis racemosa*	5	0	0	0	0	0	5
31	*Eragrostis rigidior*	48	47	0	11	0	42	148
32	*Eragrostis teichophora*	16	16	0	0	0	0	32
33	*Eragrostis viscosa*	26	5	0	0	0	33	64
34	*Erharta erecta*	0	5	0	0	0	0	5
35	*Helictotrichon turgidulum*	178	44	259	132	353	31	997
36	*Hemarthria altissima*	0	0	0	35	0	19	54
37	*Heteropogon contortus*	68	0	0	0	39	59	166
38	*Heteropogon macrostachyus*	19	0	0	0	0	9	28
39	*Heteropon contortu*	0	0	0	7	0	0	7
40	*Hyparrhenia cymbaria*	44	93	0	34	0	114	285
41	*Hyparrhenia filipendula*	100	274	39	28	19	195	655
42	*Hyparrhenia hirta*	132	161	0	0	0	103	396
43	*Hyparrhenia tamba*	20	84	0	11	75	50	240
44	*Hyperthelia dissoluta*	57	261	186	0	241	72	779
45	*Imperata cylindrica*	0	0	0	0	19	0	19
46	*Koeleria capensis*	0	0	0	0	0	8	8
47	*Monocymbium ceresiiforme*	10	0	0	18	19	60	107
48	*Panicum coloratum*	0	0	0	0	0	26	26
49	*Panicum maximum*	0	0	0	0	39	0	39
50	*Panicum natalensis*	0	0	0	12	0	6	18
51	*Panicum schinzii*	4	0	0	14	0	8	26
52	*Pennisetum macrourum*	71	0	0	0	0	80	151
53	*Pennisetum sphacelatum*	0	0	0	14	0	0	14
54	*Pentaschistis natalensis*	0	0	0	0	0	6	6
55	*Pentaschistis pallida*	0	0	0	12	0	0	12
56	*Schizachyrium jeffreysii*	0	0	0	11	14	50	75
57	*Schizachyrium sanguineum*	0	0	0	0	8	45	53
58	*Setaria pallide-fusca*	0	0	89	19	70	31	209
59	*Setaria sphacelata*	0	18	0	26	0	6	50
60	*Setaria verticillata*	0	0	0	0	0	4	4
61	*Sorghum bicolor*	0	0	0	0	0	7	7
62	*Sorghum versicolor*	0	0	0	0	0	9	9
63	*Sporobolus africanus*	0	0	0	24	35	23	82
64	*Sporobolus festivus*	0	0	0	0	0	5	5
65	*Sporobolus fimbriatus*	0	0	0	24	0	12	36
66	*Sporobolus panicoides*	0	0	0	0	0	7	7
67	*Stenotaphrum secundatum*	10	0	0	16	0	0	26
68	*Stipagrostis namaquensis*	0	0	0	0	0	84	84
69	*Themeda triandra*	42	176	14	139	23	70	464
70	*Trachypogon spicatus*	64	0	0	19	187	41	311
71	*Tragus berteronianus*	0	6	0	0	0	0	6
72	*Tristachya leucothrix*	0	0	0	15	0	13	28
73	*Urelytrum agropyroide*	8	0	0	0	13	8	29
Species richness		32	24	10	34	23	55	73
Total		1592	1653	1364	1283	1743	2606	10203

**Table 2:** Description of the vertical and horizontal structure of the six dambos assessed in the Niassa National Reserve. Variables describes vertical structure, includes the percentage of trees in the lower, middle and upper. While in the horizontal structure are abundance, dominance, frequency and Importance Value Index (IVI).

Family	N^o^	Specie	Abundance		Dominance		Frequency		% of Tree/Strata			Volume	IVI
			Ab (n)	Ar (%)	Da (m^2^/ha)	Dr (%)	Fa (%)	Fr (%)	Lower	Middle	Upper	m^3^/ha	
Anacardiaceae	1	*Ozoroa concolor*	19	0.35	0.01	0.10	6.90	0.63	0.00	84.21	15.79	0.05	1.08
	2	*Ozoroa paniculosa*	7	0.13	0.00	0.00	5.17	0.47	28.57	71.43	0.00	0.00	0.60
	3	*Ozoroa sphaerocarpa*	2	0.04	0.00	0.00	1.72	0.16	0.00	100.00	0.00	0.00	0.20
Annonaceae	4	*Annona senegalensis*	514	9.42	0.16	1.84	79.31	7.28	0.97	97.86	1.17	1.18	18.54
	5	*Antidesma venosum*	69	1.27	0.01	0.07	12.07	1.11	1.45	98.55	0.00	0.03	2.44
	6	*Artabotrys monteiroae*	12	0.22	0.03	0.29	5.17	0.47	0.00	83.33	16.67	0.24	0.98
	7	*Cleistochlamys kirkii*	1	0.02	0.00	0.02	1.72	0.16	0.00	0.00	100.00	0.02	0.20
	8	*Friesodielsia obovata*	1	0.02	0.00	0.01	1.72	0.16	0.00	100.00	0.00	0.01	0.19
	9	*Xylopia parviflora*	2	0.04	0.00	0.00	1.72	0.16	0.00	100.00	0.00	0.00	0.20
Apocynaceae	10	*Diplorhynchus condylocarpon*	272	4.99	0.10	1.12	37.93	3.48	3.31	93.75	2.94	0.63	9.59
Asteraceae	11	*Vernonia colorata*	2	0.04	0.00	0.00	1.72	0.16	0.00	100.00	0.00	0.00	0.20
Burseraceae	12	*Commiphora africana*	4	0.07	0.00	0.00	1.72	0.16	0.00	100.00	0.00	0.00	0.24
	13	*Commiphora glandulosa*	2	0.04	0.00	0.00	1.72	0.16	0.00	100.00	0.00	0.00	0.20
	14	*Commiphora harveyi*	5	0.09	0.02	0.28	1.72	0.16	0.00	40.00	60.00	0.26	0.53
	15	*Commiphora pyracanthoides*	26	0.48	0.00	0.05	3.45	0.32	0.00	100.00	0.00	0.02	0.85
Caesalpiniaceae	16	*Albizia antunesiana*	2	0.04	0.04	0.48	3.45	0.32	0.00	50.00	50.00	0.67	0.84
	17	*Albizia forbesii*	9	0.17	0.06	0.66	6.90	0.63	0.00	33.33	66.67	0.82	1.46
	18	*Albizia tanganyicensis*	7	0.13	0.03	0.40	1.72	0.16	0.00	71.43	28.57	0.47	0.69
	19	*Bauhinia petersiana*	9	0.17	0.00	0.04	1.72	0.16	0.00	100.00	0.00	0.02	0.36
	20	*Brachystegia boehmii*	4	0.07	0.09	1.07	5.17	0.47	0.00	25.00	75.00	1.32	1.62
	21	*Brachystegia spiciformis*	219	4.02	0.68	7.79	31.03	2.85	0.91	67.12	31.96	8.97	14.65
	22	*Brachystegia utilis*	97	1.78	0.21	2.46	27.59	2.53	7.22	75.26	17.53	2.61	6.77
	23	*Dalbergia melanoxylon*	5	0.09	0.00	0.01	1.72	0.16	0.00	100.00	0.00	0.01	0.26
	24	*Julbernardia globiflora*	9	0.17	0.03	0.39	8.62	0.79	11.11	44.44	44.44	0.44	1.35
	25	*Pylostygma toningii*	4	0.07	0.00	0.03	1.72	0.16	0.00	100.00	0.00	0.02	0.27
Capparaceae	26	*Boscia mossambicensis*	20	0.37	0.02	0.25	6.90	0.63	0.00	90.00	10.00	0.25	1.25
	27	*Maerua angolensis*	5	0.09	0.00	0.04	5.17	0.47	0.00	80.00	20.00	0.03	0.61
	28	*Maerua kirkii*	6	0.11	0.01	0.14	5.17	0.47	0.00	83.33	16.67	0.18	0.73
	29	*Maerua schinzii*	32	0.59	0.02	0.19	5.17	0.47	0.00	90.63	9.38	0.15	1.26
Celastraceae	30	*Gymnosporia mossambicensis*	13	0.24	0.00	0.02	5.17	0.47	0.00	100.00	0.00	0.01	0.74
	31	*Gymnosporia senegalensis*	1	0.02	0.00	0.00	1.72	0.16	0.00	100.00	0.00	0.00	0.18
	32	*Maurocenia frangula*	1	0.02	0.01	0.08	1.72	0.16	0.00	0.00	100.00	0.10	0.25
	33	*Putterlickia verrucosa*	1	0.02	0.00	0.00	1.72	0.16	0.00	100.00	0.00	0.00	0.18
Chrysobalanaceae	34	*Parinari curatellifolia*	83	1.52	0.03	0.32	17.24	1.58	0.00	98.80	1.20	0.16	3.42
Clusiaceae	35	*Garcinia livingstonei*	25	0.46	0.01	0.13	13.79	1.27	0.00	100.00	0.00	0.06	1.86
Combretaceae	36	*Combretum adenogonium*	96	1.76	0.12	1.43	20.69	1.90	0.00	78.13	21.88	1.44	5.08
	37	*Combretum apiculatum*	2	0.04	0.00	0.01	1.72	0.16	0.00	100.00	0.00	0.01	0.21
	38	*Combretum collinum*	31	0.57	0.03	0.35	18.97	1.74	3.23	90.32	6.45	0.26	2.66
	39	*Combretum molle*	1	0.02	0.00	0.00	1.72	0.16	0.00	100.00	0.00	0.00	0.18
	40	*Combretum nelsonii*	6	0.11	0.00	0.00	1.72	0.16	33.33	66.67	0.00	0.00	0.27
	41	*Combretum paniculatum*	6	0.11	0.02	0.17	3.45	0.32	0.00	66.67	33.33	0.17	0.60
	42	*Combretum psidioides*	231	4.24	0.29	3.31	70.69	6.49	1.30	80.95	17.75	2.99	14.03
	43	*Pteleopsis anisoptera*	13	0.24	0.00	0.00	1.72	0.16	23.08	38.46	38.46	0.00	0.40
	44	*Pteleopsis myrtifolia*	51	0.94	0.20	2.33	22.41	2.06	15.69	9.80	74.51	2.75	5.33
	45	*Terminalia brachystemma*	8	0.15	0.01	0.12	5.17	0.47	0.00	75.00	25.00	0.10	0.74
	46	*Terminalia gazensis*	19	0.35	0.01	0.11	5.17	0.47	0.00	94.74	5.26	0.09	0.94
	47	*Terminalia mollis*	112	2.05	0.09	1.09	22.41	2.06	2.68	84.82	12.50	0.83	5.20
	48	*Terminalia randii*	8	0.15	0.00	0.01	6.90	0.63	0.00	100.00	0.00	0.00	0.78
	49	*Terminalia sericea*	148	2.71	0.16	1.86	27.59	2.53	0.68	79.73	19.59	1.49	7.10
	50	*Terminalia zambesiaca*	1	0.02	0.00	0.01	1.72	0.16	0.00	100.00	0.00	0.01	0.19
Dipterocarpaceae	51	*Monotes glaber*	1	0.02	0.00	0.00	1.72	0.16	0.00	100.00	0.00	0.00	0.18
Ebenaceae	52	*Diospyros kirkii*	13	0.24	0.02	0.22	10.34	0.95	0.00	76.92	23.08	0.16	1.41
	53	*Diospyros lycioides*	1	0.02	0.00	0.00	1.72	0.16	0.00	100.00	0.00	0.00	0.18
	54	*Diospyros natalensis*	7	0.13	0.00	0.02	1.72	0.16	0.00	100.00	0.00	0.01	0.31
	55	*Diospyros usambarensis*	3	0.06	0.00	0.02	1.72	0.16	0.00	0.00	100.00	0.01	0.23
	56	*Diospyros villosa*	5	0.09	0.00	0.00	1.72	0.16	0.00	100.00	0.00	0.00	0.25
Euphorbiaceae	57	*Bridelia cathartica*	7	0.13	0.00	0.01	5.17	0.47	0.00	100.00	0.00	0.00	0.61
	58	*Hymenocardia acida*	101	1.85	0.03	0.34	24.14	2.22	2.97	92.08	4.95	0.20	4.41
	59	*Margaritaria discoidea*	206	3.78	0.14	1.65	12.07	1.11	2.91	91.75	5.34	0.91	6.54
	60	*Phyllanthus reticulatus*	35	0.64	0.01	0.11	10.34	0.95	5.71	85.71	8.57	0.05	1.70
	61	*Pseudolachnostylis maprouneifolia*	392	7.19	0.45	5.17	60.34	5.54	0.00	80.61	19.39	4.52	17.89
	62	*Spirostachys africana*	7	0.13	0.00	0.03	3.45	0.32	0.00	100.00	0.00	0.02	0.48
Fabaceae	63	*Burkea africana*	55	1.01	0.28	3.20	18.97	1.74	1.82	40.00	58.18	3.92	5.95
	64	*Cassia abbreviata*	4	0.07	0.00	0.03	1.72	0.16	0.00	100.00	0.00	0.01	0.26
	65	*Dichrostachys cinerea*	14	0.26	0.00	0.06	8.62	0.79	0.00	100.00	0.00	0.02	1.10
	66	*Indigofera jucunda*	10	0.18	0.00	0.04	6.90	0.63	0.00	100.00	0.00	0.01	0.85
	67	*Indigofera lyalli*	48	0.88	0.01	0.09	5.17	0.47	6.25	93.75	0.00	0.03	1.44
	68	*Mundulea sericea*	8	0.15	0.00	0.00	3.45	0.32	0.00	100.00	0.00	0.00	0.47
	69	*Pericopsis angolensis*	22	0.40	0.13	1.52	13.79	1.27	0.00	45.45	54.55	1.92	3.19
	70	*Piliostigma thonningii*	45	0.83	0.01	0.06	13.79	1.27	0.00	100.00	0.00	0.02	2.16
	71	*Pterocarpus angolensis*	11	0.20	0.00	0.01	5.17	0.47	18.18	81.82	0.00	0.00	0.69
	72	*Senna petersiana*	40	0.73	0.01	0.10	5.17	0.47	0.00	100.00	0.00	0.04	1.31
	73	*Sesbania punicea*	9	0.17	0.01	0.08	1.72	0.16	0.00	77.78	22.22	0.06	0.40
	74	*Swartzia madagascariensis*	111	2.04	0.04	0.46	24.14	2.22	1.80	96.40	1.80	0.22	4.71
Flacourtiaceae	75	*Dovyalis zeyheri*	8	0.15	0.00	0.01	3.45	0.32	0.00	100.00	0.00	0.00	0.47
	76	*Flacourtia indica*	35	0.64	0.01	0.14	12.07	1.11	0.00	85.71	14.29	0.10	1.89
Lamiaceae	77	*Vitex doniana*	121	2.22	0.06	0.72	20.69	1.90	0.83	94.21	4.96	0.61	4.84
	78	*Vitex obovata*	62	1.14	0.02	0.21	12.07	1.11	0.00	100.00	0.00	0.10	2.45
	79	*Vitex payos*	23	0.42	0.00	0.05	5.17	0.47	21.74	78.26	0.00	0.03	0.95
Loganiaceae	80	*Anthocleista grandiflora*	6	0.11	0.09	1.08	1.72	0.16	0.00	33.33	66.67	1.31	1.35
	81	*Strychnos decussata*	3	0.06	0.04	0.43	3.45	0.32	0.00	33.33	66.67	0.58	0.80
	82	*Strychnos madagascariensis*	3	0.06	0.00	0.00	24.14	2.22	0.00	100.00	0.00	0.00	2.27
	83	*Strychnos pungens*	1	0.02	0.00	0.03	1.72	0.16	0.00	100.00	0.00	0.02	0.20
Meliaceae	84	*Ekebergia capensis*	2	0.04	0.01	0.08	1.72	0.16	0.00	50.00	50.00	0.08	0.27
Mimosaceae	85	*Acacia xanthophloea*	1	0.02	0.00	0.00	1.72	0.16	0.00	100.00	0.00	0.00	0.18
	86	*Amblygonocarpus andongensis*	18	0.33	0.03	0.29	5.17	0.47	0.00	61.11	38.89	0.27	1.10
Moraceae	87	*Ficus nigrescensis*	2	0.04	0.06	0.73	1.72	0.16	0.00	0.00	100.00	0.80	0.92
	88	*Ficus sycomorus*	2	0.04	0.11	1.24	1.72	0.16	0.00	0.00	100.00	1.65	1.43
Myrtaceae	89	*Syzygium cordatum*	607	11.13	3.87	44.49	58.62	5.38	0.99	43.66	55.35	53.53	61.00
	90	*SYzygium guineense*	171	3.14	0.37	4.24	18.97	1.74	3.51	78.95	17.54	4.92	9.12
Olacaceae	91	*Ximenia americana*	4	0.07	0.00	0.03	3.45	0.32	0.00	100.00	0.00	0.02	0.42
	92	*Ximenia caffra*	12	0.22	0.01	0.11	5.17	0.47	0.00	91.67	8.33	0.07	0.80
Pittosporaceae	93	*Pittosporum viridiflorum*	6	0.11	0.00	0.03	1.72	0.16	0.00	100.00	0.00	0.02	0.30
Proteaceae	94	*Faurea saligna*	12	0.22	0.01	0.13	3.45	0.32	0.00	66.67	33.33	0.11	0.66
	95	*Protea nitida*	604	11.07	0.05	0.62	15.52	1.42	6.29	93.71	0.00	0.22	13.12
Rhamnaceae	96	*Ziziphus mucronata*	3	0.06	0.00	0.01	3.45	0.32	0.00	100.00	0.00	0.00	0.38
Rubiaceae	97	*Vangueria cyanescens*	8	0.15	0.00	0.03	3.45	0.32	0.00	100.00	0.00	0.02	0.49
	98	*Burchellia bubalina*	30	0.55	0.06	0.66	6.90	0.63	0.00	63.33	36.67	0.75	1.85
	99	*Canthium gilfillanii*	10	0.18	0.00	0.03	3.45	0.32	0.00	100.00	0.00	0.01	0.53
	100	*Crossopteryx febrifuga*	207	3.80	0.12	1.43	41.38	3.80	1.93	89.37	8.70	1.03	9.02
	101	*Feretia aeruginescens*	3	0.06	0.00	0.01	1.72	0.16	0.00	100.00	0.00	0.00	0.22
	102	*Gardenia ternifolia*	17	0.31	0.00	0.03	12.07	1.11	0.00	100.00	0.00	0.01	1.45
	103	*Keetia gueinzii*	35	0.64	0.04	0.48	13.79	1.27	0.00	77.14	22.86	0.46	2.39
	104	*Lagynias lasiantha*	2	0.04	0.00	0.02	1.72	0.16	0.00	100.00	0.00	0.01	0.22
	105	*Pavetta zeyheri*	15	0.28	0.00	0.05	5.17	0.47	0.00	100.00	0.00	0.03	0.80
	106	*Vangueria infausta*	8	0.15	0.00	0.02	6.90	0.63	0.00	100.00	0.00	0.01	0.80
Rutaceae	107	*Ptaeroxylon obliquum*	11	0.20	0.01	0.16	6.90	0.63	0.00	90.91	9.09	0.17	1.00
Sapindaceae	108	*Dodonaea angustifolia*	5	0.09	0.00	0.01	1.72	0.16	0.00	100.00	0.00	0.00	0.26
Sapotaceae	109	*Manilkara mochisia*	13	0.24	0.00	0.02	3.45	0.32	0.00	100.00	0.00	0.01	0.57
Vitaceae	110	*Rhoicissus tridentata*	6	0.11	0.00	0.00	1.72	0.16	0.00	100.00	0.00	0.00	0.27
TOTAL			5454	100.00	8.70	100.00	1089.66	100.00	NA	NA	NA	107.91	300.00

Note: Absolute (Ab) and Relative (Ar) Abundance, Absolute (Ad) and Relative (Dr) Dominance, Absolute (Fa) and Relative (Fr) Frequency, Importance Value Index (IVI).

**Fig. 1:**
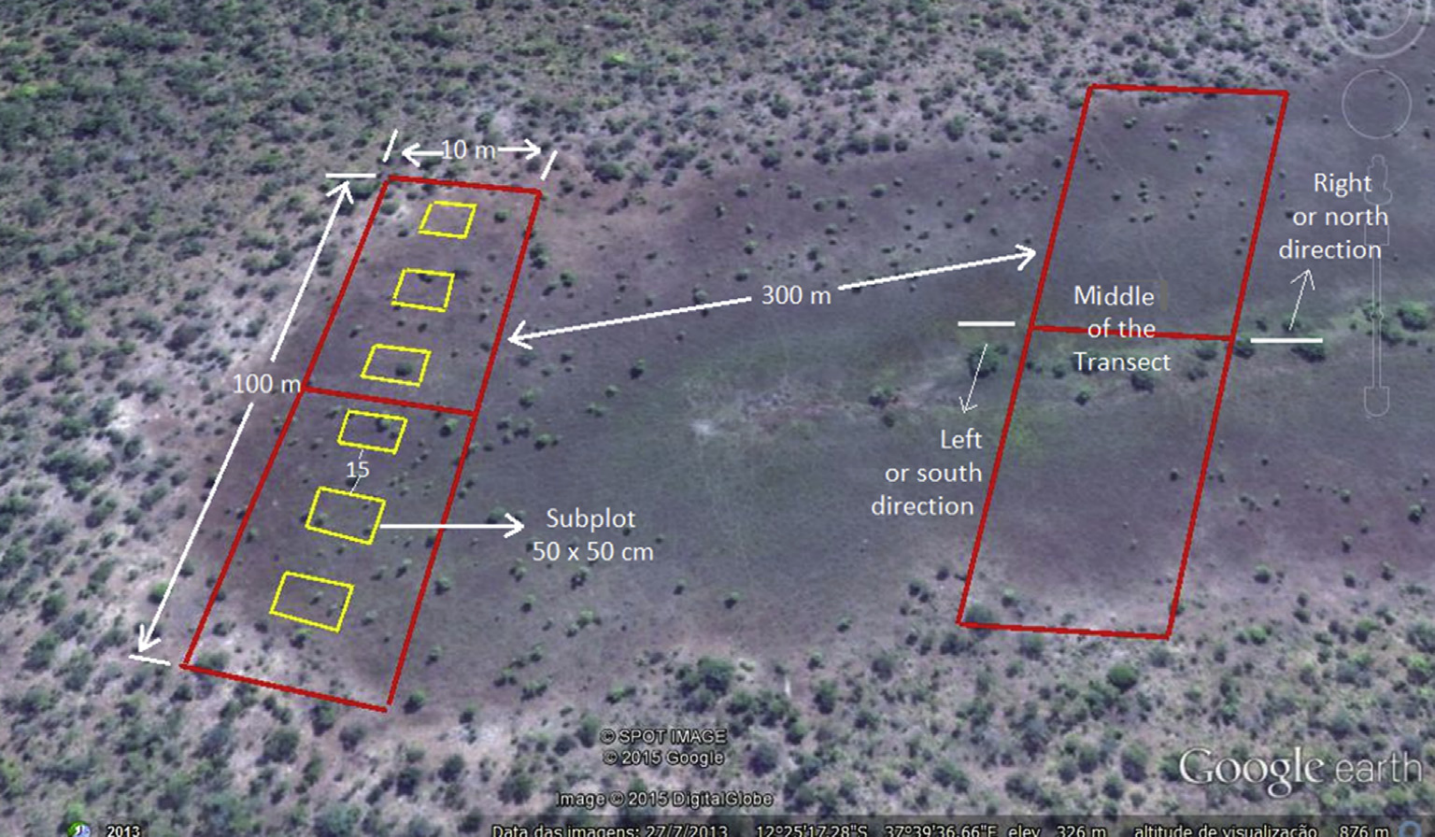
Illustration of the sampling procedure in the dambos of the Niassa National Reserve (Mozambique). Mean transects, for woody vegetation survey are placed perpendicularly to the length of the dambo every 300 m (primary units in red). Subplots (secondary units in yellow), for grass vegetation survey, are located inside each mean transect and spaced apart approximately 15 m.

Table 3 presented the location and the general characterization of all dambos assessed. While in the Fig. 1, represents the sampling scheme used to collect data on trees, shrub and grass and vegetation In the main transects and subplots respectively.

**Table 3:** Location and characterization of the dambos assessed in the Niassa National Reserve, northern Mozambique.

COORDINATES					
Dambo	Location	Latitude S	Longitude E	Elevation (m)	Characteristics
1	Mbatamila center	12°10′48.60″	37°32′19.0″	451	Sc and Sw
2	Kiboko	12°25′50.81″	37°40′11.97″	284	Sc and Sw
3	Kuchiranga	12°25′09.52″	37°39′57.22″	290	Sc and Pw
4	Nyate Junction	12°08′26.72″	37°34′41.63″	450	Pw
5	Matondovela Junction (10 km from Mbatamila)	12°08′19.34″	37°32′05.31″	421	Af and Pw
6	Matondovela Junction (25 km from Mbatamila)	12°09′06.53″	37°28′13.78″	482	Af and Pw

Characteristics: Sc – shifting cultivation, Sw – seasonal water, Pw – permanent water, Af – artisanal fishing.

## 2 Experimental design, materials and methods

The six dambos sampled in this study were selected using Google Earth and MODIS satellite images. After the identification of the dambos, an exploratory field trip was made to verify whether the candidate dambos were appropriate for the establishment of the survey plots. The selection was required to offer a representative sample size and proximity to the Mbatamila Center Office of the Reserve, due to budget constraints and poor road access. A preliminary, basic characterization was conducted in each dambo, which consisted of assessing the occurrence of fire in the last two years, shifting cultivation (Sc), artisanal fishing (Af), soils and vegetation characteristics, seasonal water (Sw) or permanent water (Pw). Table 3 presents detailed information regarding the location and characteristics of the selected dambos. The occurrence of grass vegetation was dominant in all dambos, which is a defining characteristic of these ecosystems, as described in the literature [3]. Fire also occurred in all dambos in the recent years.

Data were collected using a two-stage systematic sampling procedure. In the first stage, tree and shrub vegetation information was collected in transects of 100 × 10 m (0.1 ha), established perpendicular to the length of the dambo. The center of the plot was marked after identifying the bottom of the dambo in the middle of the transect, extending 50 m to each side. Because the distance between transects within the dambos was 300 m, the number of transects established in each dambo varied, depending on the dambo's size. In total, 58 transects were established and surveyed. In a second stage, grass vegetation was counted, height measured and collected for later identification in six square subplots of 0.25m^2^ (50 × 50 cm), established within the main transects, according to Tito et al. (2009) [4]. The distance between each pair of subplots was about 15 m. Thus, a total of 336 subplots were established in all transects (see Fig. 1).

The tree heights were measured with the support of a hypsometric bar and Vertex when necessary, whereas the diameters (dbh and D) were measured with a measuring tape. Subsequently, each stem was identified to species and family and recorded in the field, based on authoritative field guides to of trees of southern Africa [5] and the grasses of southern Africa [6]. For the species that were difficult to identify in the field, samples were collected for later identification by a botanist.

The successional stage of each species in the vertical structure was analyzed according to its position, by dividing the forest canopy in three main strata, namely: lower, middle and upper, based on the variable height (h), according to the following equation: lower (us) $h_{j}<\left(\bar{h}-S\right)$, middle (ms) $\left(\bar{h}-S\right)\leq h_{j}<\left(\bar{h}+S\right)$ and upper (ls) $h_{i}\geq \left(\bar{h}+S\right)$, where $\bar{h}$ is the mean height of all trees in a given sample, S is the standard deviation of *h* in a given sample and $h_{i}$ is the total height of j-th individual tree. According to Hosokawa et al. (2008) [1], a given species is well placed in the forest when it is well represented in all forest strata, with a large proportion of trees in the lower stratum. More information regarding the data collection and analysis is provided in Mbanze et al. (2019) [7].

## References

[1] Hosokawa R., Moura J.B., Cunha U. (2008). Introduç∼ ao ao maneio e economia de florestas.

[2] Krebs C.J. (2014). Ecological data for field studies. Ecol. Methodol..

[3] Whitlow R. (1990). Conservation status of wetlands in Zimbabwe: past and present. Geojournal.

[4] Tito M., León M., Porro R. (2009). Guia para la determinación de carbono en pequenãs propriedade rurales. http://www.worldagroforestry.org/.

[5] van Wyk B., van Wyk P. (1997). Field Guide to Trees of Southern Africa.

[6] van Oudtshoorn F. (2018). Guide to Grasses of Southern Africa. http://www.briza.co.za.

[7] Mbanze A.A., Martins A., Rivaes R., Ribeiro-Barros A., Ribeiro N. (2019). Vegetation structure and effects of human use of the dambos ecosystem in northern Mozambique. Glob. Ecol. Conserv..

